# Experiences of ethnic-racial discrimination, self-esteem, and subjective well-being in the Peruvian population

**DOI:** 10.3389/fpsyg.2026.1777639

**Published:** 2026-04-14

**Authors:** Andy Rick Sánchez-Villena, Isabella Temple-Focón

**Affiliations:** 1 Vicerrectorado de Investigación, Universidad Señor de Sipán, Chiclayo, Peru; 2 Facultad de Ciencias de la Salud, Carrera Profesional de Psicología, Universidad Privada del Norte, Trujillo, Peru; 3 Forum, Centro de Estudios Familiares, Cajamarca, Peru

**Keywords:** discrimination, ethnicity, mental health, race, racism

## Abstract

**Introduction:**

Ethnic-racial discrimination can have adverse effects on the physical health and psychological wellbeing of those who experience it. Peru is a country where the perception of discrimination based on race/ethnicity is high. However, few psychological studies have addressed this issue. In this regard, the aim of this study is to examine the relationship between experiences of ethnic-racial discrimination, self-esteem, and subjective wellbeing in a Peruvian sample.

**Material and methods:**

Using a cross-sectional associative design, a structural regression model was created with R software, in which experiences of ethnic-racial discrimination predict self-esteem and subjective wellbeing, and these are correlated. To this end, 645 Peruvian adults (65.1% women and 34.9% men) between the ages of 18 and 63 (*M* = 24.97; SD = 8.02) were included.

**Results:**

The model showed adequate fit for the overall sample (CFI = 0.990; TLI = 0.989; RMSEA = 0.072; SRMR = 0.059), indicating that experiences of discrimination decrease self-esteem (β = −0.111) and subjective well-being (β = −0.087; *p* = 0.076); however, the effects were small. In addition, the model was found to be invariant across gender and age. At the descriptive level, men and younger individuals experienced greater effects.

**Conclusions:**

Experiences of ethnic and racial discrimination can lower self-esteem, but their effects are minor. Similarly, with subjective wellbeing, however, the effects are almost null. This relationship is more pronounced in males and younger individuals.

## Introduction

Peru is one of the countries with the highest perception of ethnic-racial discrimination (ERD) in Latin America, which is reflected in the results of a number of surveys. Thus, it has been reported that 46.5% of respondents believed that Peruvian society is very racist; 68% believe that discrimination is a current problem and that it is promoted by the media (Sánchez-Villena and Temple-Focón, 2023), and the most recent results indicate that 50% of Peruvians have perceived ERD due to their phenotype, socioeconomic status, educational level, and culture (Instituto Nacional de Salud Mental Honorio Delgado—Hideyo Noguchi, 2025; Ministerio de Cultura, 2018). Regarding differences based on gender and age, the results are inconsistent as a higher prevalence has been reported in women than in men, especially among younger women (Instituto Nacional de Estadística e Informática, 2011), while other studies suggest that it is men who experience greater discrimination regardless of age (Sulmont, 2005). Furthermore, the latest statistics show that Peruvian men face greater discrimination than women (Instituto Nacional de Estadística e Informática, 2024).

ERD is conceptualized as the differential treatment of people belonging to ethno-racial groups (Kirkinis et al., 2021), which is expressed through negative attitudes toward those with different cultures or phenotypes, or through the restriction of public spaces, mockery, direct aggression, and even the use of humor (Breazu and Machin, 2022). Furthermore, it is considered a social and public health problem as it affects the wellbeing of those who experience it, as it has adverse physical and psychological implications (Cave et al., 2020; Dean and Thorpe, 2022; Priest et al., 2024).

In fact, systematic reviews, meta-analyses, and empirical studies provide evidence that experiences of discrimination are associated with greater psychological distress, depression, anxiety, suicide risk, risky sexual behaviors, substance use, loneliness, feelings of humiliation, and a sense of injustice; lower academic performance and engagement, lower self-esteem, and subjective wellbeing (Becerra et al., 2020; Benner et al., 2018; Bottiani et al., 2020; Cave et al., 2020; Cénat et al., 2024; Civitillo et al., 2024; Coimbra et al., 2022; Gabarrell-Pascuet et al., 2023; Kirkinis et al., 2021; Pascoe et al., 2022; Priest et al., 2024; Urzúa et al., 2018). Specifically, the last two factors are particularly relevant, as they are often the most frequently reported in review studies and are robust predictors of depression, anxiety, and suicidality, mainly (Coimbra et al., 2022; Espinosa, 2021; Jang et al., 2014; Kim et al., 2023; Soto-Sanz et al., 2019; Urzúa et al., 2019; Yang et al., 2019).

Given their importance for psychological adjustment and mental health, it is necessary to consider how these constructs are conceptualized within the psychological literature. In this context, self-esteem is defined as the perception that people have of their own worth, dignity and competence (Monteiro et al., 2022); whereas subjective wellbeing refers to each individual's assessment of their life and their satisfaction with it (Ventura-León et al., 2025).

However, there is a lack of research examining the relationship between experiences of discrimination, self-esteem, and subjective wellbeing in the Latin American context in general, and in Peru in particular. In fact, the gap in knowledge about the psychological impact of racism and discrimination in the Peruvian context has been reported by a systematic review (Sánchez-Villena and Temple-Focón, 2025).

Only hypotheses have been put forward that racism can diminish self-esteem, happiness, and life satisfaction (Alarcón, 2011; León, 2010) given that unfair treatment on racial grounds generates personal offense, which in turn causes emotional pain (Bruce, 2019; Fossa, 2009; Rosas, 2021). Furthermore, the few psychological studies that have analyzed the psychological impact of ethnic-racial discrimination in the Peruvian context have indicated that it is linked to depression, anxiety, propensity to anger, suicidal ideation (Sánchez-Villena and Temple-Focón, 2023), neuroticism, and dark personality traits (Sánchez-Villena and Temple-Focón, 2024).

Therefore, this research fills a theoretical gap, providing evidence to support the idea that ERD is a relevant factor in mental health, so that the fight against discrimination in public policies can be improved and given greater importance. In this sense, the aim is to examine the relationship between experiences of ethnic-racial discrimination, self-esteem, and subjective wellbeing in a Peruvian sample. In view of the above, it is hypothesized that (a) experiences of ethnic-racial discrimination have a negative effect on subjective wellbeing and, similarly, that (b) experiences of ethnic-racial discrimination have a negative effect on the self-esteem of the Peruvian population.

## Materials and methods

### Study design

A quantitative, cross-sectional, associative study was conducted to determine the relationship between variables (Ato et al., 2013).

### Participants

A total of 715 individuals participated, responding to an online form shared on social media. A non-probability snowball sampling method was used (Sedgwick, 2013), as a link was shared through Facebook, Instagram, WhatsApp and LinkedIn, asking those who would like to participate to share it on their own social media networks so that participants could be referred by others. Four participants were excluded due to incomplete forms and 66 due to being underage (< 18). Therefore, the sample consisted of 645 Peruvian citizens (65.1% women and 34.9% men) between the ages of 18 and 63 (*M* = 24.97; SD = 8.02). Table 1 describes the sociodemographic data of the participants.

**Table 1:** Sociodemographic characteristics of the participants.

Characteristics	*n*	%
Age^*^	24.97	±8.02
Schooling		
Postgraduate (master's or doctorate)	44	6.8
Secondary school	67	10.4
Technical superiority	39	6.0
College graduate	495	76.7
Ethnic identity		
Aymara	2	0.3
White	97	15.0
Mixed race	437	67.8
Amazonian native	3	0.5
Black/Brown/Zambo/Mulatto/Afro-Peruvian or Afro-descendant	35	5.4
Nikkei/Tusán/Descendant of Asian population	2	0.3
Belonging to or part of another community	25	3.9
Quechua	44	6.8
Employment status		
Study and work at the same time	230	35.7
None of the above	23	3.6
Just study	284	44.0
Only works	108	16.7
Age group		
Adult (25+ years)	231	35.8
Young (18–24 years)	414	64.2

Age^*^ is a numerical variable.

### Instruments

#### Discrimination experiences scale

This instrument was originally designed by Krieger et al. (2005) whose original structure consists of a one-dimensional model with nine items designed to measure experiences of discrimination in different contexts (e.g., when seeking medical care, applying for loans or seeking assistance from the authorities). The response options are Likert-type with four categories ranging from Never = 0 to Four or more times = 3. There is also an abbreviated version proposed by Janevic et al. (2015), which considers only the most everyday situations (e.g., on the street, at school or in shops) and has the same number and categories of response options. In this study, the short version was used, which has been validated in Peru by Sánchez-Villena and Temple-Focón (2023), who assessed content validity for linguistic adjustments, internal structure validity using confirmatory factor analysis (CFI = 0.97; SRMR = 0.06), and reliability was demonstrated by the internal consistency method using the omega coefficient, yielding a value of ω = 0.80. It should be noted that the choice of the short version has practical reasons, as the use of short tests reduces participant fatigue and is efficient in contexts where multiple instruments are used, thereby improving the response rate (Cooper, 2023; Ghafourifard, 2024); furthermore, the five-item test focuses on common social interaction contexts, enabling the assessment of forms of discrimination that occur in daily life.

#### Rosenberg self-esteem scale

This is a self-report instrument with 10 items and 4 response options (1 = strongly disagree, 2 = disagree, 3 = agree, and 4 = strongly agree), whose objective is to measure self-esteem. It was originally developed by Rosenberg (1965). In Peru, the instrument has been adapted to different populations and a two-dimensional structure has been reported (Ventura-León et al., 2018), a one-dimensional structure with control of the effect on reverse items (Sánchez-Villena et al., 2021), and even a one-dimensional version with only direct items (Vilca et al., 2022). For the present research, the 10-item direct version will be used, whose reliability showed an ω = 0.96.

#### Subjective well-being scale

This is a self-report instrument with a single dimension and three items with seven response options (strongly disagree = 1 to strongly agree = 7), which aims to measure subjective well-being and was developed by Su et al. (2016). Psychometric properties indicate that it is a unidimensional scale with adequate reliability (α = 0.872). In Peru, internal structural validity was demonstrated using psychometric networks, indicating unidimensionality (CFI = 1.00, RMSEA = 0.000), and validity in relation to other variables showed that the scale is negatively correlated with depression (*r* = −0.44) and anxiety (*r* = −0.34). Reliability was assessed using structural consistency, with a SC coefficient of 1 (Ventura-León et al., 2025).

### Procedure

The research began with a request for review and approval from the Ethics Committee. Once the letter of approval had been issued, data collection commenced via an online form, the link to which was shared on social media (Facebook, WhatsApp, Instagram and LinkedIn) between January and April 2023. This internet-mediated method has been shown to have the advantage of reducing social desirability bias, particularly when addressing topics of a certain degree of sensitivity, such as experiences of sexual harassment, as it increases the level of anonymity and confidentiality (Latkovikj and Popovska, 2019).

The first page of the form displayed an informed consent agreement, which offered the option to agree to participate in the study and answer the questions in the questionnaires, or to close the form to end their participation. The consent form contained information regarding the name of the principal investigator, the aim of the research, potential risks and benefits, as well as the use of the data collected; in this regard, the ethical principles established in international and national declarations, such as the Declaration of Helsinki and the code of the Peruvian College of Psychologists (Colegio de Psicólogos del Perú), were complied with.

Subsequently, the database was downloaded in.xlsx format for use in Excel and then imported into the R software, where the data were analyzed using a structural equation modeling approach with the lavaan package; a structural regression model was run as the variables were latent (Feng and Hancock, 2024) and this model was compared by gender and age to determine whether the model is invariant and whether there are effects at the regression level.

This last variable was categorized into two groups: young people (aged 18–24) and adults (aged 25 and over), based on the classification proposed by Mansilla (2000) for Peru.

### Data analysis

This was carried out using R software version 4.2.2 with the lavaan package. First, empty cells (*n* = 4) were removed and data relating to minors (*n* = 66) was filtered out. Next, a structural equation model was created using the WLSMV estimator, due to the ordinal nature of the variables. To determine the adequacy of the model, the cut-off points of CFI > 0.95; TLI > 0.95; RMSEA < 0.08 and SRMR < 0.06 (Hu and Bentler, 1999) were taken into account. Finally, the invariance of the model according to gender and age (vital cicle) was checked by examining the configural, metric, and strong equivalence (Pendergast et al., 2017), for which the cut-off points of ΔCFI < −0.01 and ΔRMSEA ≥ −0.01 (Chen, 2007) were considered.

It is important to note that, for the purposes of the age-invariant analysis, two categories were created—young people and adults—based on the age reported by the participants. To this end, the cut-off points of 18–24 for young people and 25 and over for adults were used, as proposed by Mansilla (2000) for the Peruvian context.

### Ethical considerations

This study was approved by the ethics committee of the Universidad Privada del Norte (No. 0004-2023/ID-CIEI) and the data protection officer of the University of Lleida (Spain), as the ethical guidelines established by the Peruvian College of Psychologists, Peruvian Data Protection Law No. 29733, Spanish Organic Law 3/2018 on personal data protection and digital rights, and the Declaration of Helsinki were followed at all times.

## Results

Table 2 shows the correlation between variables, where it can be seen that experiences of discrimination are inversely related to subjective wellbeing (Rho = −0.079) and self-esteem (Rho = −0.106). However, in terms of effect size, the correlation is low in both cases.

**Table 2:** Correlation matrix between variables.

Variables	SWB	EAR	EOD
SWB	1		
EAR	0.627	1	
EOD	−0.079	−0.106	1

SWB, subjective wellbeing; EAR, self-esteem; EOD, experiences of discrimination.

### Structural regression

The proposed model showed an excellent fit for the general sample (CFI = 0.990; TLI = 0.989; RMSEA = 0.072; SRMR = 0.059), for women (CFI = 0.992; TLI = 0.991; RMSEA = 0.072; SRMR = 0.060), men (CFI = 0.985; TLI = 0.983; RMSEA = 0.085; SRMR = 0.085), young people (CFI = 0.992; TLI = 0.991; RMSEA = 0.071; SRMR = 0.058), and adults (CFI = 0.987; TLI = 0.985; RMSEA = 0.072; SRMR = 0.070).

### Invariance

The invariance of the proposed model was verified according to gender and age. The results indicate that the data correspond adequately to the structure regardless of both variables (see Table 3).

**Table 3:** Invariance according to gender and age.

Variable	Invariance	CFI	TLI	RMSEA	SRMR	ΔCFI	ΔRMSEA
Gender	Configural	0.990	0.988	0.077	0.068	-	-
	Metric	0.990	0.988	0.078	0.071	−0.001	0.001
	Strong	0.990	0.990	0.070	0.069	0.001	−0.008
Age	Configural	0.990	0.990	0.071	0.062	-	-
	Metric	0.990	0.991	0.067	0.065	0.000	0.001
	Strong	0.990	0.992	0.064	0.064	0.000	0.001

**Figure 1:**
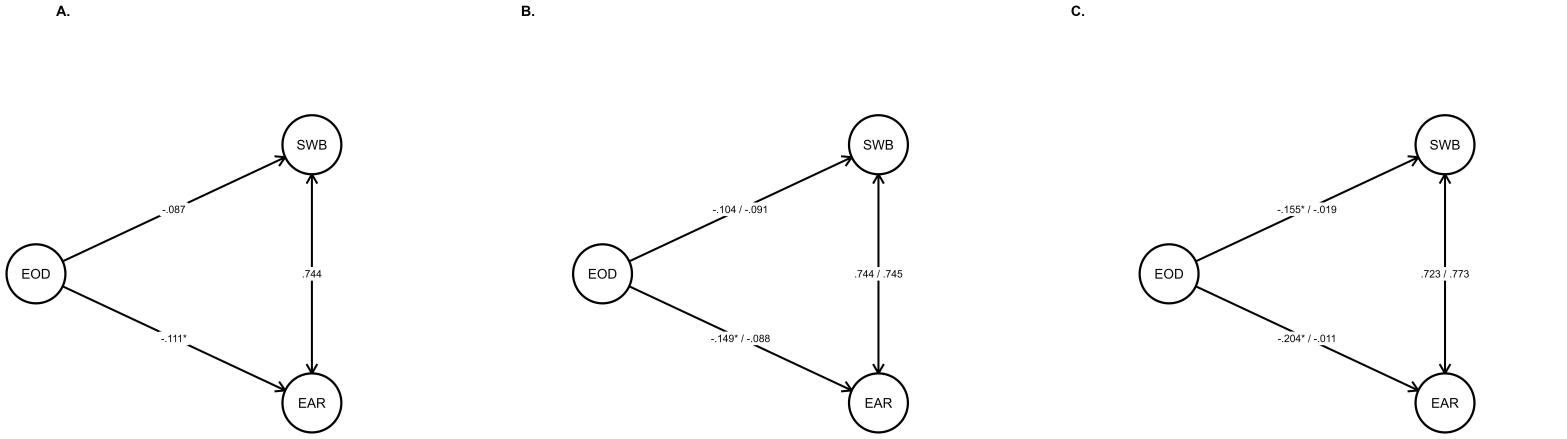
Structural regression models. Item loadings have been omitted to improve the visibility of the regressions. **(A)** Structural model in overall sample. **(B)** Structural model by gender (men on the left, women on the right). **(C)** Structural model by age (young people on the left, adults on the right). *Statistically significant.

Figure 1 indicates that, for the general sample, experiences of ethnic-racial discrimination are associated with lower self-esteem (β = −0.111; *p* = 0.027), an effect that is statistically significant, and with lower subjective wellbeing (β = −0.087; *p* = 0.076), although this latter association does not reach statistical significance; both effect sizes are small.

The results are similar when considering gender. Among men ethnic-racial discrimination is negatively associated with self-esteem, reaching statistical significance, and with subjective wellbeing, although the latter association is not statistically significant (β_self − esteem_ = −0.149; *p* = 0.042; β_wellbeing_ = −0.104; *p* = 0.147). Similarly among women (β_self − esteem_ = −0.088; *p* = 0.165; β_wellbeing_ = −0.091; *p* = 0.153), both associations are negative but do not reach statistical significance. Overall effect sizes are small, although the magnitude of the associations appears greater among men.

Regarding age differences, a similar pattern emerges. Among young people, ethnic-racial discrimination shows statistically significant negative associations with both self-esteem and subjective wellbeing (β_self − esteem_ = −0.204; *p* = 0.001; β_wellbeing_ = −0.155; *p* = 0.012). In contrast, among adults, no statistically significant associations are observed (β_self − esteem_ = −0.011; *p* = 0.885; β_wellbeing_ = −0.019; *p* = 0.784). Overall, although effect sizes are small, the magnitude of the associations is greater among young people than among adults.

## Discussion

Experiences of racial discrimination are social stressors that negatively impact the mental health of those who have experienced them. Peru is one of the countries with the highest perception of racism and discrimination based on ethnicity and race; however, few studies have analyzed its relationship with psychological aspects. In this regard, this study aims to explore the relationship between experiences of racial discrimination, self-esteem, and subjective wellbeing.

The results showed that within the studied sample, experiences of discrimination are significantly associated with lower levels of self-esteem and subjective wellbeing. This suggests that individuals who report higher exposure to ERD, tend to report poorer psychological outcomes. Although the observed effect sizes were small, their relevance should not be underestimated. In contexts such as Peru, where experiences of ERD are highly prevalent (Instituto Nacional de Salud Mental Honorio Delgado—Hideyo Noguchi, 2025; Ministerio de Cultura, 2018) even small associations can have meaningful implications at the population level. Furthermore, the chronic nature of exposure to ERD may lead to sustained psychological effects over time, which are not fully captured by cross-sectional studies.

These findings are consistent with previous studies conducted in contexts such as United States and Canada with second-generation migrants, university students and members of the African-descendant community (Becerra et al., 2020; Cénat et al., 2024; Espinosa, 2021) which have documented similar associations between exposure to racism and discrimination and mental health indicators. However, these comparisons should be interpreted with caution, as the sociocultural dynamics and historical trajectories, and manifestations of racism could differ from those in the Peruvian context, because in Peru, ethnic-racial discrimination is linked to colonial legacies and phenotypic hierarchies (Sánchez-Villena and Temple-Focón, 2025) which may shape its psychological impact in distinct ways.

In this sense, the results contributes to addressing a gap in Latin American context, specifically in Peru by providing empirical evidence on the relationship between ERD and psychological well-being and self-esteem which is consistent with previous studies that have indicated that exposure to racisms and ERD increase emotional sensibility, feelings of worthlessness, guilt, sadness and hopelessness, affecting the image they have of themselves and promoting feelings of unhappiness (Urzúa et al., 2018). In fact, evidence from Peru has shown that ERD takes the form of teasing and offensive comments which lead to low self-esteem and increase the risk of developing symptoms of depression, anxiety and suicidal ideation (Sánchez-Villena and Temple-Focón, 2023).

With regard to differences based on gender and age, the models showed invariance; however, there is a notable difference in the effects of ERD experiences on self-esteem and subjective wellbeing in the male group and in the younger group. These findings are consistent with the limited existing evidence from Peru, which suggests that the ERD may have a stronger impact on men and younger people (Instituto Nacional de Estadística e Informática, 2024; Sulmont, 2005). However, it differs from previous studies conducted in other contexts, which have reported stronger effects among women (Becerra et al., 2020) and adults (Cénat et al., 2024).

One possible explanation for this phenomenon is that men and young people are more exposed to situations of ERD because they have a greater presence in public spaces, which increases the likelihood of experiencing racism and ethnic-racial discrimination (Sulmont, 2005). In addition, men are more likely to attribute discriminatory treatment to ethnic-racial reasons (Benner et al., 2018). Likewise, in foreign contexts, it has been observed that women tend to receive more positive messages about their belonging to ethnic-racial groups, which increases their ethnic-racial pride and identity; therefore, this may serve as a protective factor against racist events. However, this issue has not been studied in Peru, which presents an opportunity to investigate its moderating role in future research (Sánchez-Villena and Temple-Focón, 2023).

This research is not exempt from limitations, four of which stand out. The first is the sampling strategy, since non-probabilistic sampling reduces the generalizability of the results. The second is that the sample consists mainly of people who self-identify as mestizo; consequently, it was not possible to compare relationships according to ethnic identity or to identify potential differences between the various ethnic groups. It would therefore be necessary for the different ethnic groups to be equally represented in order to identify potential differences in the effects of experiences of ethnic and racial discrimination, as the type and intensity of discrimination may differ across groups.

The third is the cross-sectional design, which does not allow us to understand the dynamics of the variables over time. The fourth, is the measurement of ERD experiences based on a single instrument, as this is intersectional and may be accompanied by other forms of discrimination, such as sexism, homophobia, etc. Consequently, it is recommended that future studies consider longitudinal designs with probabilistic sampling and that measurement instruments for other types of discrimination be implemented. In addition, it is suggested that other variables that play a mediating or moderating role, such as pride and ethnic identity as well as coping strategies, be incorporated.

Despite these limitations, the research has the strength of being one of the first in Latin America to address the effects of ERD on psychological aspects and to empirically demonstrate its harmfulness to people's wellbeing, especially that of young people and men. It is therefore necessary for the fight against discrimination to take a differentiated approach based on sex and age. Finally, the study emphasizes the need to include interventions that reduce the impact of social stressors in order to promote better mental health in Peru, as racism and discrimination remain highly prevalent and ongoing phenomena.

Furthermore, the results underscore the need to develop culturally sensitive interventions aimed at mitigating the impact of social stressors such as discrimination, taking into account the specific forms and meanings that racism adopts in the Peruvian context.

In conclusion, the findings suggest that experiences of ERD are associated with lower levels of self-esteem and subjective wellbeing, with stronger associations observed among men and younger individuals. However, further studies are needed for the results to be conclusive.

## Data Availability

The datasets presented in this article are not readily available because the results are derived from the doctoral thesis of the first author, whose data are protected. However, the data may be requested with adequate justification. Requests to access the datasets should be directed to andysavi92@gmail.com.
